# Neural mechanism facilitating PM2.5-related cardiac arrhythmias through cardiovascular autonomic and calcium dysregulation in a rat model

**DOI:** 10.1038/s41598-023-41148-8

**Published:** 2023-09-25

**Authors:** Tsung-Ying Tsai, Li-Wei Lo, Wei-Lun Lin, Yu-Hui Chou, Wen-Han Cheng, Shin-Hui Liu, Cheryl C. H. Yang, Terry B. J. Kuo, Shih-Ann Chen

**Affiliations:** 1 Heart Rhythm Center, Division of Cardiology, Department of Medicine, Taipei Veterans General Hospital, 201, Sec. 2, Shih-Pai Road, Taipei, Taiwan; 2 National Yang Ming Chiao Tung University, Taipei, Taiwan; 3 Cardiovascular Center, Taichung Veterans General Hospital, Taichung, Taiwan; 4 Institute of Clinical Medicine and Cardiovascular Research Institute, National Yang Ming Chiao Tung University, Taipei, Taiwan; 5 Institute of Biomedical Science, Mackay Medical College, New Taipei city, Taiwan; 6 Institute of Brain Science, National Yang Ming Chiao Tung University, Taipei, 112 Taiwan; 7 Tsoutun Psychiatric Center, Ministry of Health and Welfare, Nantou, Taiwan; 8 National Chung Hsing University, Taichung, Taiwan

**Keywords:** Cardiovascular biology, Environmental impact

## Abstract

Particulate matter < 2.5 μm (PM2.5) exposure is associated with increased arrhythmia events and cardiovascular mortality, but the detailed mechanism remained elusive. In the current study, we aimed to investigate the autonomic alterations in a rodent model after acute exposure to PM2.5. Twelve male WKY rats were randomized to control and PM2.5 groups. All were treated with 2 exposures of oropharyngeal aerosol inhalations (1 μg PM2.5 per gram of body weight in 100 μL normal saline for the PM2.5 group) separately by 7 days. Polysomnography and electrocardiography were surgically installed 7 days before oropharyngeal inhalation and monitored for 7 days after each inhalation. Physiologic monitors were used to define active waking (AW), quiet sleep (QS), and paradoxical sleep (PS). Autonomic regulations were measured by heart rate variability (HRV). The protein expression of ventricular tissue of the 2 groups was compared at the end of the experiment. In sleep pattern analysis, QS interruption of the PM2.5 group was significantly higher than the control group (0.52 ± 0.13 events/min, 0.35 ± 0.10 events/min, *p *= 0.002). In HRV analysis, the LF/HF was significantly higher for the PM2.5 group than the control group (1.15 ± 0.16, 0.64± 0.30, *p* = 0.003), largely driven by LF/HF increase during the QS phase. Ionic channel protein expression from Western blots showed that the PM2.5 group had significantly lower L-type calcium channel and higher SERCA2 and rectifier potassium channel expressions than the control group, respectively. Our results showed that acute PM2.5 exposure leads to interruption of QS, sympathetic activation, and recruitment of compensatory calcium handling proteins. The autonomic and calcium dysregulations developed after PM 2.5 exposure may explain the risk of sleep disturbance and sleep-related arrhythmia.

## Introduction

Particulate matter < 2.5 μm (PM2.5) exposure is the most important environmental risk factor in the world^1,2^. Acute exposure to PM2.5 is associated with increased arrhythmia events, including atrial fibrillation, ventricular dysrhythmia, and arrhythmia hospital visits^3–5^. Acute exposure to PM2.5 is also strongly associated with other cardiovascular events, including acute myocardial infarction, stroke, and cardiovascular mortality^6–9^. Although the association between PM2.5 exposure and cardiovascular risk is undeniable, the causality between exposure and events cannot be established with epidemiology studies alone.

In human studies, PM2.5 has demonstrated a plethora of effects on the cardiovascular system, including inflammation, autonomic modulation, prothrombotic state, and endothelial dysfunction^10^. The impact of acute PM2.5 exposure on autonomic modulation is of particular importance as this may be the fastest reaction after acute exposure^11^. In addition, cardiovascular events occur most frequently in the early morning, at the time of circadian autonomic alteration^12^. The alteration of autonomic function after PM2.5 exposures are demonstrated in studies of heart rate variability (HRV) and blood pressure in humans^13^. Our group has previously demonstrated the diurnal change of autonomic modulation after acute PM2.5 exposure as well as its impact on arrhythmia burden^14,15^. However, the observed changes in the cardiovascular system are the result of the complex interaction between PM2.5 and many other associated factors. Thus, an animal study is warranted to establish the causal link between PM2.5 exposure and cardiac autonomic alteration. In this study, we aimed to investigate autonomic alterations and sleep patterns in a rodent model after acute exposure to PM2.5.

## Methods

### Animal preparation and study design

**Figure 1:**
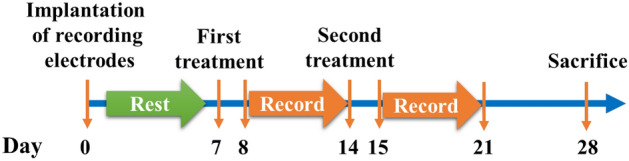
Experiment protocol of the study. All rats received head circuit installation on day 1. On day 7 (first treatment) and day 14 (second treatment), aerosol exposures were carried out in both groups. Six rats were given treatments with PM2.5-saline aerosol [1 μg(PM2.5)/1 g (body weight) in 100 μL normal saline each time] via tracheal injection, while 6 control rats were treated with 100 μL saline. Physiologic monitoring was conducted in the following 24 h after each treatment. The rats were sacrificed on day 28.

The study protocol was reviewed and approved by the Institutional Animal Care and Committee of Taipei Veterans General Hospital (IACUC Number: 2020-157). All animal preparations and experiment methods were conducted in accordance with ARRIVE guidelines and local regulations. The experiments were conducted on twelve 8- to 10-week-old Wistar-Kyoto (WKY) rats. The rats were obtained from BioLASCO. They were raised in a sound-attenuated room with a 12:12 light–dark cycle (lights on from 08:30 AM to 08:30 PM) and at an appropriate temperature (22 ± 2 °C) and humidity (40–70%) control. Food and water were provided ad libitum. The rats were randomized into the control group and the PM2.5 group. All rats received head circuit installation as described below on day 1. On day 7 (first treatment) and day 14 (second treatment), aerosol exposures were carried out in both groups. Six rats were treated with PM2.5-saline aerosol [1 μg (PM2.5)/1 g (body weight) in 100 μL normal saline each time] via tracheal injection, while 6 control rats were treated with 100 μL saline. Physiologic monitoring was conducted in the following 24 h after each treatment. Figure 1 shows the experimental protocol of this study.

### Head circuit installation and signal recording procedures

All animals were implanted in the electroencephalogram (EEG) recording electrodes on day 1 under anesthesia. The detailed surgical procedures were described in previous publications^16–18^. Under pentobarbital anesthesia (50 mg/kg, intraperitoneally), each rat was placed in a standard stereotaxic apparatus where electrodes for a parietal EEG, nuchal electromyogram (EMG), and electrocardiogram (ECG) were implanted. Two stainless steel screws were driven bilaterally into the skull overlaying the parietal cortex (2 mm posterior to and ± 2 mm lateral to the bregma), and a reference electrode was implanted 2 mm caudal to the lambda. Two seven-strand stainless steel microwires were inserted into the dorsal neck muscles to record the electromyogram (EMG). ECG was recorded via a pair of microwires placed dorsally under the skin. (Supplemental Fig. 1) After surgery, the rats were individually housed in translucent cages for a 1 week recovery and subsequent experiment.

### Experimental protocol

**Figure 2:**
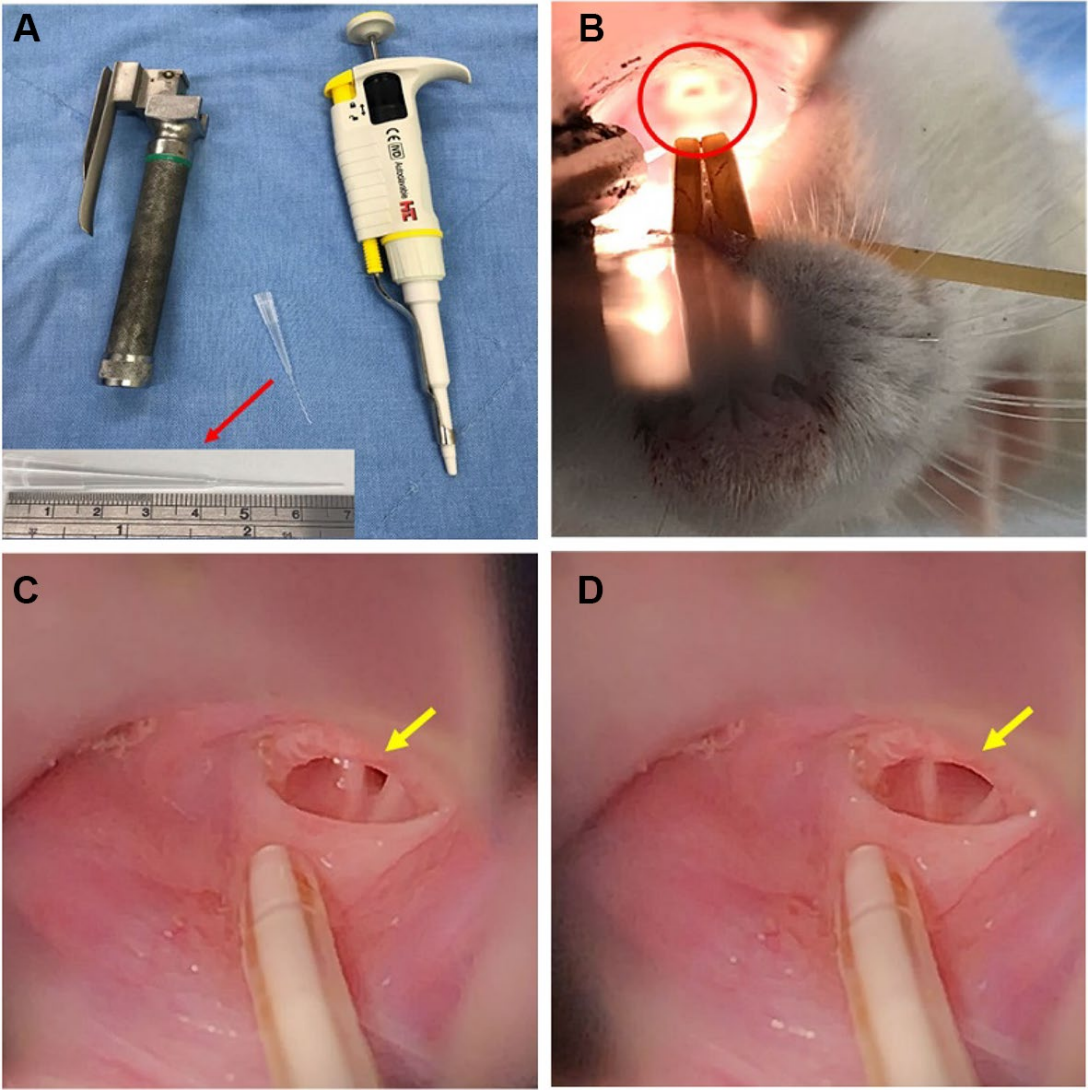
Exposure procedure of the animals. (**A**) laryngoscope and micropipette for tracheal injection, (**B**) directly visualize the vocal cord (red circle) with the laryngoscope, (**C**) no injection when the vocal cord is in close position (yellow arrow), (**D**) tracheal injection was performed when the vocal cord is in open position (yellow arrow).

All rats received the experiment 1 week after head circuit installation for proper wound healing. In brief, male WKY rats were anesthetized with pentobarbital (50 mg/kg, intraperitoneally). We then visualized the vocal cord with a small animal laryngoscope. We performed tracheal injection of 100 μL saline aerosol solution or pure saline was given via a micropipette each time when the vocal cords were in an open position (Fig. 2)^19,20^. After the exposure, we waited for 24 h before collecting sleep cycles and cardiac autonomic activity using wireless transmission of polysomnography and EVG for a duration of 7 days. At the end of the experiment, rats were euthanized by exsanguination under anesthesia on day 28, and heart tissues were harvested.

### Data acquisition and measurements

**Figure 3:**
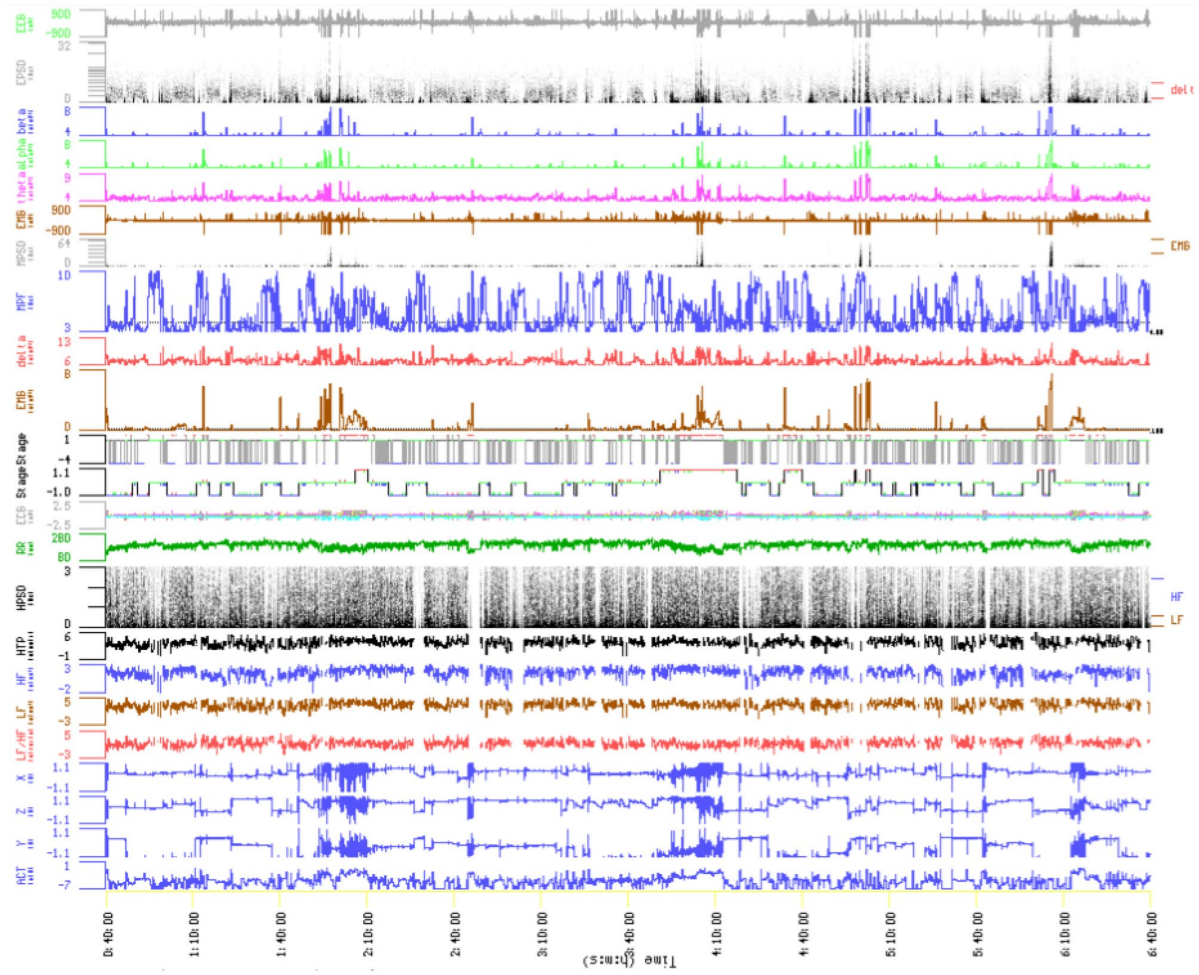
An example output of EEG, EMG and ECG. The example output of the EEG, EMG, and ECG recording from the data of the control group. Channel 1–5 and 9 shows the EEG recording related, channel 6–8 and 10 show the EMG recording, while channel 13–19 shows the ECG related. The speed of recording is 64 Hz thus, 6 h seconds of data was recorded in this output. *EEG* electroencephalogram, *EMG* electromyography, *ECG* electrocardiography.

The electrophysiological signals were recorded by a wireless sensor (Size: 25 × 21 × 13 mm, weight: 8.6 g, KY4C, K&Y Lab, Taiwan) developed by our laboratory, and the performance of the telemetry system has been validated^16,21^. The EEG, EMG, and ECG signals were amplified at 1000, 1000, and 500-folds, respectively, and filtered at 0.16–48 Hz, 34–103 Hz, and 0.72–103 Hz, respectively. The EEG, EMG, and ECG signals were synchronously digitized by an analog–digital converter at different sampling rates (125, 250, and 500 Hz, respectively). The digitized signals were then wirelessly transmitted to a digital data recorder (KY3, K&Y Lab) at a radio frequency of 2.4 GHz. All digitized data were stored in a flash memory card for subsequent offline analysis. An example figure of the EEG, EMG, and ECG output is shown in Fig. 3.

### Sleep pattern analysis

We defined sleep–wake stages via EEG and EMG records. Continuous power spectral analysis was applied to the EEG and EMG signals using a Hamming window of 16 s (50% overlap), from which the mean power frequency (MPF) of the EEG and the power magnitude of the EMG were quantified. For each time epoch (8 s), the conscious state was defined as active waking (AW) if the corresponding MPF and EMG powers were above a pre-defined MPF threshold (TMPF) and EMG power threshold (TEMG), respectively, as QS if the MPF and EMG power were below the TMPF and the TEMG, respectively; and as paradoxical sleep (PS) if the MPF was above the TMPF and the EMG power was below the TEMG. If the MPF was below the TMPF and the EMG power was above the TEMG, an erroneous epoch was identified and excluded from the analysis.

To define the threshold, first, the recorded data were cut into 5 h segments. Second, the 5 h time series of MPF underwent a histogram analysis, from which 2 separate populations that were respectively related to the AW/PS complex and QS could be identified^16^. Therefore, the TMPF could be set to discriminate these 2 populations. Third, the histogram of the EMG time series also had 2 populations, which were respectively related to AW and the QS/PS complex. Therefore, TEMG could be set to discriminate these 2 populations. Fourthly, the TMPF and TEMG were manually fine-tuned by an experienced rater. A sleep–wake stage was formed when there were at least 6 consecutive identical epochs and an interruption was marked when consecutive epochs were less than 6. To quantify the architecture of sleep–wake stages, the time (total time of a given stage within the analysis period) and duration (average bout duration of a given stage) were calculated. The interruption rate heightened meant deep sleep was disturbed.

We quantified the δ power (1–4 Hz), θ power (4–8 Hz), α power (8–13 Hz) and β power (13–32 Hz) of the EEG spectrogram based on fast Fourier transformation^22^. Δ and β powers were used as a measure to evaluate depth of sleep. An augmentation of δ power and a suppression of β power indicated increased sleep depth^23^. Θ wave and hippocampal activity were relationships during both active exploratory behavior and REM sleep^24^. Α wave activity often appeared in REM sleep and semi-arousal state, and it was common in chronic fatigue patients and may amplify the effects of other sleep disorders^25^. Sleep quality was assessed by the δ power of the QS stages and the interruptions of the QS and PS stages. In addition, stage transitions were also identified and enumerated (AW to QS transition, A–Q; AW to PS transition, A–P; QS to AW transition, Q–A; QS to PS transition, Q–P; PS to AW transition, P–A; PS to QS transition, P–Q). Stage transitions provide information about the tendency of a given stage to transition to another stage.

### Heart rate variability (HRV) analysis

The R–R interval was estimated continuously from the digitized electrocardiogram signals. The R–R interval was resampled and interpolated at 64 Hz to provide continuity in the time domain and then was truncated into 16 s time segments (1024 points) with 50% overlap. These sequences were analyzed by fast Fourier transform after the application of a Hamming window. The frequency domain HRV parameters, including the high-frequency power (HF, frequency range: 0.6–2.4 Hz) and the low-frequency power (LF, frequency range: 0.06–0.6 Hz), provided estimates for cardiac vagal and cardiac sympathetic modulation, respectively^26^. The LF/HF ratio showed the balance between the sympathetic and parasympathetic nervous systems.

### Protein expression analysis

The rats were sacrificed at the end of the experiment (day 28), frozen ventricular tissues were homogenized, and proteins were isolated for protein expression analysis. Using western blot analysis, we evaluated the ion channel expression levels. The ionic channel proteins included cardiac calcium channels CaV1.2 (Thermo Scientific, Waltham, MA, USA), SR-calcium-ATPase2 (SERCA2, Thermo Scientific, Waltham, MA, USA), ryanodine receptor (RyR) 2 (Abcam, Cambridge, CB2 0AX, UK), and sodium/calcium-exchanger (NCX, Swant, Bellinzona, Switzerland), cardiac sodium channels Nav1.5 (Alomone Labs, Jerusalem, Israel), voltage-gated potassium channel Kv11.1 (Alomone Labs, Jerusalem, Israel), and KvLQT1 (Merck Millipore, Darmstadt, Germany in USA), inward-rectifier potassium ion channel Kir2.1 (Santa Cruz, CA, USA). The samples were harvested after finishing the experiment and flushed free of blood. Every part of the tissue was stored at − 80 °C until assay. The myocardium sample was placed in 1 mL lysis buffer (20 mM Tris–HCL [pH 7.4], 150 mM NaCl, 1% Nonidet P-40, 1 mM EDTA, 1 mM phenylmethylsulfonyl fluoride, 50 mM NaF, 10 μg/mL antipain, 10 μg/mL leupeptin, 10 μg/mo aprotinin, and 1 mM sodium vanadate), homogenized intermittently at 4 °C for 90 s, and incubated for 2 h. Thereafter, the sample was centrifuged at 15,000*g* for 20 min. Equivalent amounts of protein were mixed with loading buffer (10% β-mercaptoethanol, 0.004% bromophenol blue, 0.25 M Tris–HCl, pH 6.8, 4% SDS, and 10% glycine), boiled for 10 min, and loaded onto a 16% gradient SDS–polyacrylamide gel. The proteins were transferred to PVDF membranes in the presence of glycine transfer buffer (0.25 M Tris base, 1.92 M glycine, and 1% SDS). The PVDF membranes were blocked with 2% albumin in TBS-T buffer (20 mM Tris–HCl, pH 7.6, 137 mM NaCl, and 0.05% Tween-20) for 30 min at room temperature. The membranes were subsequently incubated overnight with the primary antibody in 2% albumin with TBS-T at 4 °C. Excess primary antibody was washed from the PVDF membranes with three 10 min washes in TBS-T, and these membranes were then incubated with the ECL anti-rabbit IgG fragment in TBS-T. After 3 further 10 min washes in TBS-T, bound antibodies were detected using the western blotting detection system^27^.

### Statistical analysis

Data were presented as mean ± standard deviation. The distribution data were checked for normal distribution with The Shapiro–Wilk and compared between the PM2.5 and control groups with the Student’s t-test and Mann–Whitney U Test for normally and non-normally distributed parameters, respectively. The sleep pattern and EEG data were compared between the PM2.5 and control groups. The HRV data, including the RR interval, LF/HF, LF, and HF, were logarithmically transformed to correct the skewness of the distribution and compared with the Student’s t-test^28^. The protein expressions were also compared between the PM2.5 and the control groups using the Student’s t-test. Statistical significance was assumed for *P* < 0.05. All statistical analyses were carried out with SPSS 25.0 software (IBM, Inc. Chicago, IL, USA).

### Ethical approval

The study was approved by the Institutional Animal Care and Committee of Taipei Veterans General Hospital (IACUC number: 2020–157).

## Results

### Sleep analysis results

**Figure 4:**
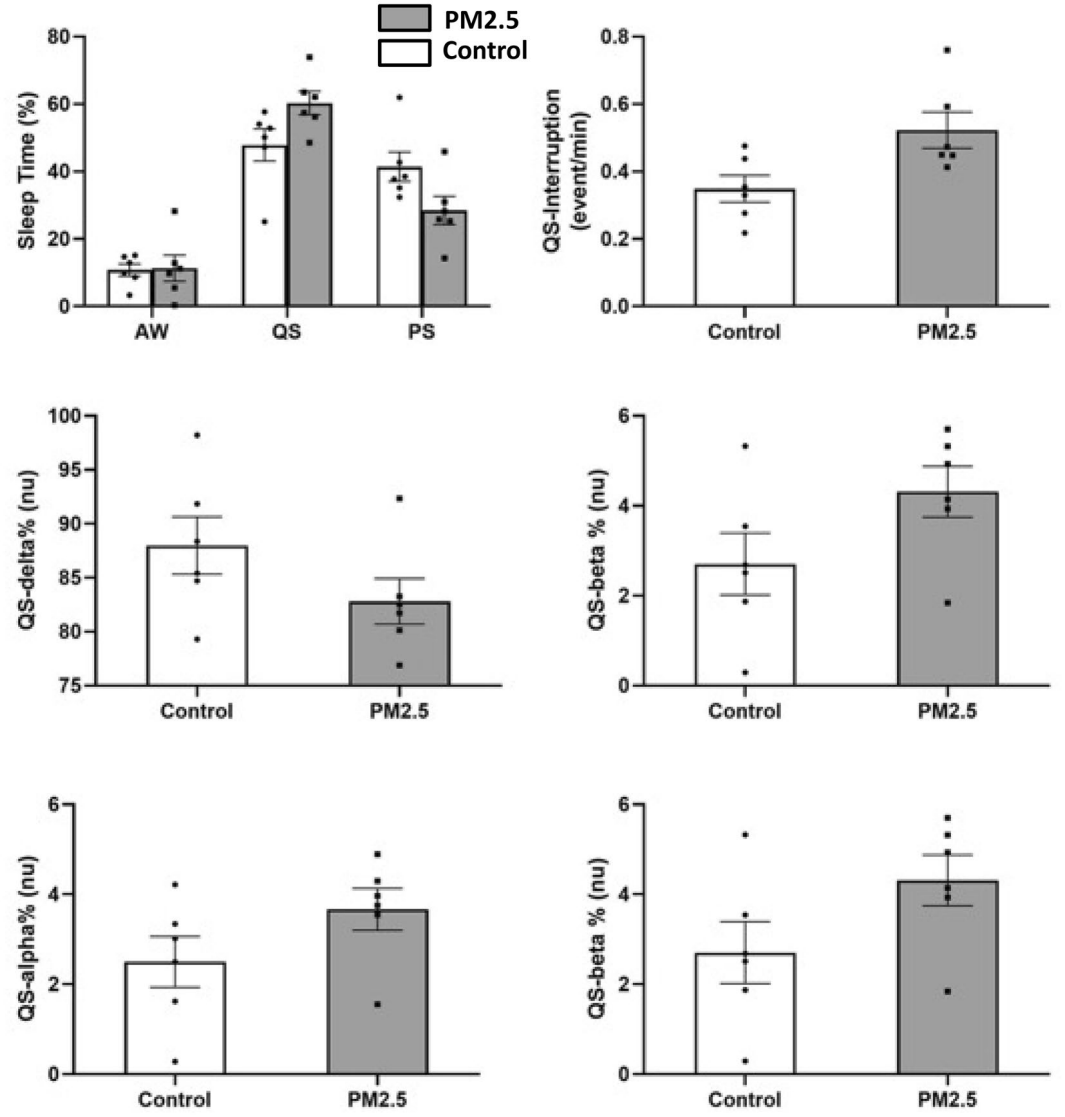
Heart rate variability analysis of the PM2.5 group (n = 6) and the control group (n = 6). Panel A showed the LF/HF ratio in different sleep stages. Panel B showed the R-R interval in different sleep stages. Panel C showed the LF HRV in different sleep stages. Panel D showed the HF HRV in different sleep stages. *Indicate *p* < 0.05 using the Student’s t-test. *HF* High frequency power (frequency range: 0.6–2.4 Hz), *LF* low frequency power (frequency range: 0.06–0.6 Hz), *AW* active waking, *PS* paradoxical sleep, *QS* quiet sleep.

The sleep pattern analysis is demonstrated in Fig. 4 and Table 1. In the sleep pattern analysis, we found that the duration of QS, and AW in PM2.5 group were numerically lower, and the PS duration was longer, when compared to those in the control group, respectively (Table 1). In QS architecture analysis, the interruption rate was significantly higher in PM2.5 group, when compared to the control group (0.52 ± 0.13, 0.35 ± 0.10, *p* = 0.002). The α, β, and θ waves during QS were numerically lower for the PM2.5 group, while their δ wave was numerically higher when compared to those in the control group, respectively.

**Table 1:** Sleep structure analysis of PM2.5 group and control group.

	Control	PM2.5	p value
AW (%)	10.76 ± 4.47	11.29 ± 9.45	0.904
QS (%)	47.84 ± 11.69	60.30 ± 8.50	0.060
PS (%)	41.40 ± 10.66	28.41 ± 10.27	0.057
Interruption (event/min)	0.35 ± 0.10	0.52 ± 0.13	0.002
QS-delta% (nu)	87.98 ± 6.51	82.81 ± 5.19	0.159
QS-theta% (nu)	6.82 ± 3.51	9.21 ± 2.94	0.229
QS-alpha% (nu)	2.50 ± 1.39	3.67 ± 1.14	0.140
QS-beta% (nu)	2.70 ± 1.68	4.31 ± 1.39	0.100

*AW* active waking, *PS* paradoxical sleep, *QS* quiet sleep, *nu* normalized unit;

*p* value of student’s t-test.

### HRV analysis results

**Figure 5:**
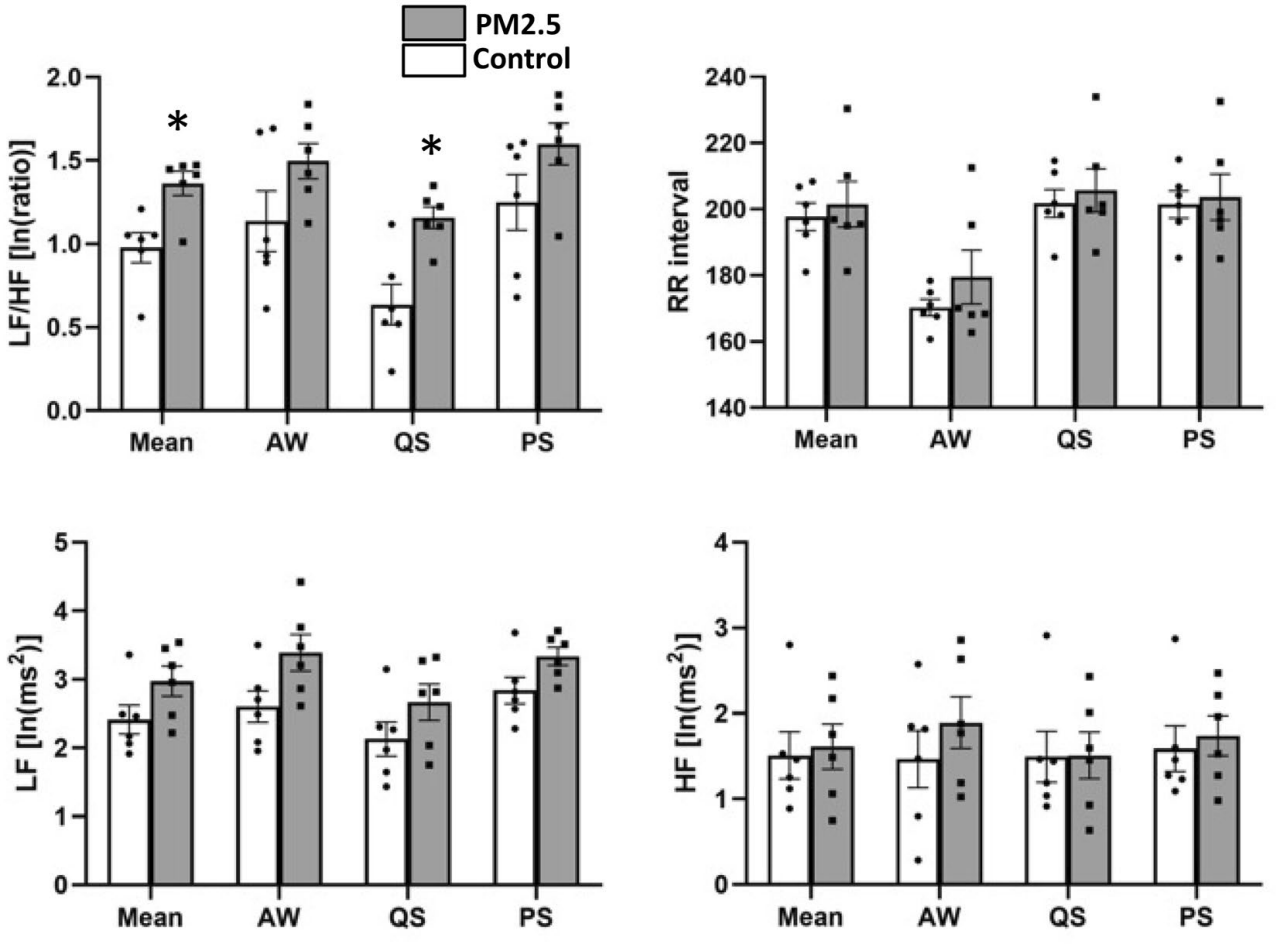
Sleep analysis of the PM2.5 group (n = 6) and the control group (n = 6). Panel A showed the time in different sleep stages of the 2 groups. Panel B showed the QS interruption rate of the 2 groups. Panel C through F showed the EEG analysis of the 2 groups in QS stage. *Indicate *p* < 0.05 using the Student’s t-test. *AW* active waking, *PS* paradoxical sleep, *QS* quiet sleep.

The HRV analysis results are demonstrated in Fig. 5 and Table 2. In the HRV analysis, we found that the LF/HF ratio was significantly higher for the PM2.5 group (1.36 ± 0.18 vs. 0.90 ± 0.26, *p* = 0.004) when compared to the control group. This was largely driven by LF/HF increase during the QS phase (1.16 ± 0.16 vs. 0.64 ± 0.30, *p* = 0.003) while the LF/HF were similar between both groups in AW (1.50 ± 0.26 vs. 1.14 ± 0.44, *p* = 0.117) and PS (1.60 ± 0.30 vs. 1.25 ± 0.41, *p* = 0.124) periods. The RR interval, LF, and HF were similar between both groups, respectively. The higher LF/HF was mainly driven by a higher LF of the PM2.5 group, especially in the QS phase, when compared to the control group.

**Table 2:** Heart rate variability analysis of PM2.5 group and control group.

	Control	PM2.5	*p* value
Mean			
RR interval (ms)	197.68 ± 10.18	201.51 ± 16.82	0.644
HF (ms^2^)	1.51 ± 0.67	1.61 ± 0.65	0.794
LF (ms^2^)	2.41 ± 0.52	2.97 ± 0.53	0.092
LF/HF	0.90 ± 0.26	1.36 ± 0.18	0.005
AW			
RR interval(ms)	170.26 ± 6.15	179.50 ± 19.82	0.818^a^
HF (ms^2^)	1.47 ± 0.82	1.89 ± 0.74	0.365
LF (ms^2^)	2.60 ± 0.56	3.39 ± 0.65	0.049
LF/HF	1.14 ± 0.44	1.50 ± 0.26	0.117
QS			
RR interval(ms)	201.77 ± 10.34	205.67 ± 16.11	0.628
HF (ms^2^)	1.49 ± 0.73	1.51 ± 0.67	0.969
LF (ms^2^)	2.13 ± 0.60	2.67 ± 0.64	0.167
LF/HF	0.64 ± 0.30	1.16 ± 0.16	0.004
PS			
RR interval(ms)	201.44 ± 10.09	203.67 ± 16.98	0.787
HF (ms^2^)	1.59 ± 0.65	1.74 ± 0.57	0.682
LF (ms^2^)	2.84 ± 0.48	3.34 ± 0.32	0.059
LF/HF	1.25 ± 0.41	1.60 ± 0.30	0.124

*HF* high frequency power (frequency range: 0.6–2.4 Hz), *LF* low frequency power (frequency range: 0.06–0.6 Hz), *AW* active waking, *PS* paradoxical sleep, *QS* quiet sleep.

^a^*p* value from Mann–Whitney U Test. The remaining p value from Student’s t-test.

### Protein expression analysis results

**Figure 6:**
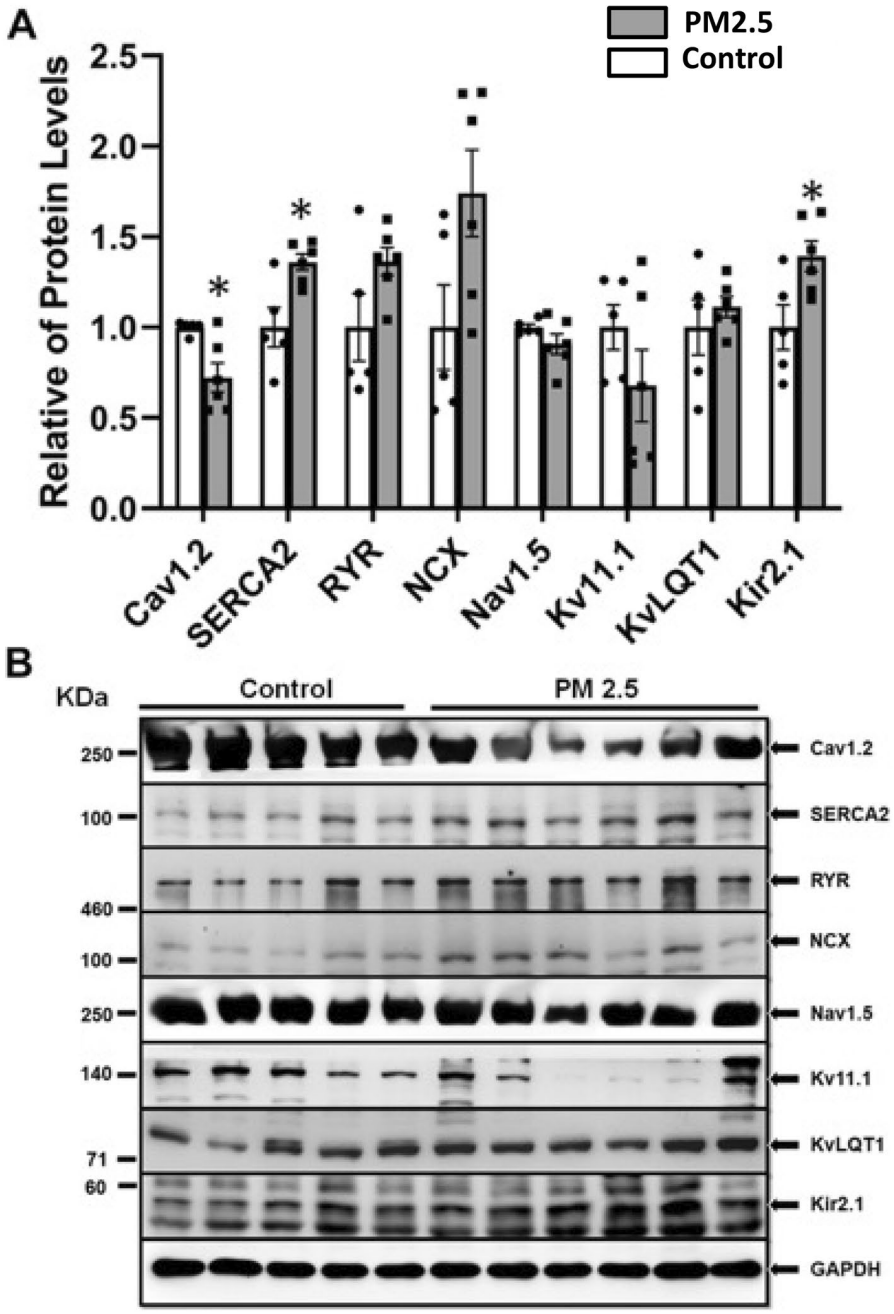
Ion channel protein expression of the ventricular tissue of the PM2.5 group (n = 6) and the control group (n = 5). Panel A showed the relative protein levels of Cav1.2, SERCA2, RYR, NCX, Nav1.5, Kv11.1, KvLQ1 and Kir2.1. Panel B showed the western blot analysis of the ion channel protein expression. * Indicate *p* < 0.05 comparison of CaV1.2 was performed with Mann–Whitney U Test, while the remaining were performed with Student’s t-test. CaV1.2 = cardiac calcium channels, SERCA2 = SR-calcium-ATPase2, RYR = ryanodine receptor, NCX = sodium/calcium-exchanger, Nav1.5 = cardiac sodium channels Nav1.5, Kv11.1 = voltage-gated potassium channel Kv11.1, KvLQT1 = voltage-gated potassium channel KvLQT1, Kir2.1 = inward-rectifier potassium ion channel.

Protein analysis is demonstrated in Fig. 6 and Supplemental Figs. 2–4. Our results showed that the PM2.5 group had significantly lower CaV1.2 (0.72 ± 0.20 vs. 1.00 ± 0.04, *p* = 0.017) and higher SERCA2 (1.36 ± 0.11 vs. 1.00 ± 0.24, *p* = 0.002) and Kir2.1 (1.39 ± 0.20 vs. 1.00 ± 0.28, *p* = 0.013) expressions when compared to those in the control group, respectively. The expression of RYR, NCX, Nav1.5, Kv11.1, and KvLQT1 channels were similar between both groups, respectively.

## Discussion

### Main findings

In the current study, we observed that an acute PM2.5 exposure induces the following changes in rats: 1. decreased QS time and increased the sleep interruption, 2. sympathetic activation and 3. Alterations of the calcium and potassium channels expression. Our results suggest that an acute PM2.5 exposure causes autonomic alteration, electrophysiological alteration and sleep structure change which may help explain the increased risk of cardiovascular events after acute exposures.

### PM2.5 exposure and sleep structure

Air pollution has been associated with reduced sleep duration and incidence of sleep disordered breathing^29,30^. PM2.5 exposure is also associated with the severity of sleep disordered breathing and increased sleep fragmentation^31,32^. Sleep fragmentation and sleep disordered breathing is a strong risk factor for many cardiovascular diseases including hypertension, atrial fibrillation, myocardial infraction, heart failure and stroke^33^. Our study is the first to demonstrate that PM2.5 exposure induces sleep fragmentation and reduced quite sleep duration in a controlled animal model. This demonstrated a direct relationship between exposure and sleep structure change without the interference of noise, light pollution, stress, and comorbidities in human observations.

### PM2.5 exposure and autonomic modulation

In previous studies, we demonstrated that acute PM2.5 exposure is associated with sympathetic activation in a diurnal pattern and that PM2.5 exposure is associated with increased arrhythmia burden^14,15^. These findings are consistent with many other human observations^34,35^. In the current study, we confirm the activation of sympathetic system in rats after acute exposure to PM2.5. We found that the LF/HF was significantly higher for PM2.5 group and especially during the quiet sleep phase. Wang et al.^36^, in another rat model study, reported that PM2.5 exposure induces heart rate variability changes, elevated blood pressure and systemic inflammation. However, the previous studies didn’t explore the association between the autonomic alteration after air pollution exposure and its interaction with sleep phases. Our results showing the concomitant sympathetic activation during QS suggests that PM2.5 exposure has a triggering effect in this particularly vulnerable phase^37^. The diurnal effect of PM2.5 exposure may be the result of complex interaction between the autonomic tone at different stages of the animal’s circadian rhythm and the effect of PM2.5 exposure, which may not manifest immediately, on different autonomic pathways, such as the muscarinic acetylcholine receptors (mAChRs), beta-adrenergic receptors and non-adrenergic, non-cholinergic (NANC) neurotransmitters such as Neuropeptide Y (NPY) and galanin^38^. The summation of these interactions is the inappropriate sympathetic activation at QS, a period when the parasympathetic activity is expected to be dominant. Further research is warranted to elucidate the mechanisms through which PM2.5 exposure affects autonomic balance and to explore potential interventions to mitigate these effects, especially during vulnerable sleep phases.

### PM2.5 exposure and protein expression

In the current study, we found a significant increase in the expression of SERCA2 and delayed rectifier potassium channel Kir2.1 alongside a reduction in L-type calcium channel (CaV1.2) expression in the rat myocardium following acute exposure to PM2.5. The upregulation of SERCA2, which has been documented in prior studies, is perceived as an acute stress response to enhance cardiac contractility^38–40^. However, it is crucial to recognize that excessive activation of SERCA2 is arrhythmogenic. The overexpression of SERCA2a improves calcium handling but results in an increased sarcoplasmic reticulum (SR) calcium load^41^. Some of the consequences SERCA overexpression are as follows: (1) SERCA2a overexpression may improve Ca^2+^ handling but at the cost of increased SR calcium load. (2) Overexpressing SERCA alters the balance between the major calcium-handling proteins, as demonstrated in the decreased L-type calcium channel of our results. The larger SR calcium store will initially lead to an increase in the calcium transient, autoregulation is ensured by (1) more rapid inactivation of subsequent calcium currents and, therefore, (2) reduced calcium entry through L-type calcium channels. The net effect is to reduce transsarcolemmal calcium flux while maintaining a normal systolic transient. Increases in SERCA protein abundance result in an increased SERCA2a load. The SERCA Ca^2+^ overload may produce spontaneous Ca^2+^ releases and thereby lead to ectopic activity. Elevated intracellular Ca^2+^ may also close gap junctions, decreasing cell-to-cell coupling, and thereby decreasing action potential conduction directly provoking arrhythmias^42,43^. The combination of these protein expression suggest that the rats’ myocardium has undergone an adaptive change after acute exposure of PM2.5. However, while the adaptations are physiological to cope with acute stress, they increase the risk for arrhythmia. Furthermore, it is important to consider the potential interaction between these adaptive changes and other regulatory proteins, such as the regulator of G protein signaling 4 (RGS4), which is abundant in the conduction tissues of the heart and is involved in the regulation of cardiac cholinergic receptor activation^44^. RGS4 also plays a significant role in protecting against arrhythmia by suppressing the pro-arrhythmogenic calcium signaling of Gq/11 protein-coupled receptors and inhibition of inflammation effect of NLRP3 inflammasome^45^.

### Study limitation

In the current study, we did not perform a subsequent experiment on cardiac myocytes, which would further link these protein expression differences to cellular dysfunction. Dong et al. showed that acute PM2.5 exposure resulted in an increased level of intracellular free Ca^2+^ in cardiomyocytes of a rodent model^46^. In a murine cardio-myocyte model, in-vivo experiment and sophisticated bioinformatics analysis revealed that PM2.5 exposure induces: (1) ROS generation, (2) dysfunction in the homeostasis of Ca2^2+^, (3) dysfunction of mitochondria, (4) reduced synthesis of ATP, and (5) Impaired cardiac-myocyte movement^47^. In the next phase of our animal studies, we plan to perform isolated cardiomyocyte experiments to obtain this important information.

## Conclusion

Our results showed that PM2.5 exposure leads to interruption of QS, sympathetic activation, and recruitment of compensatory calcium handling proteins. The autonomic and calcium dysregulations developed after PM 2.5 exposure may increase the risk of sleep-related arrhythmia.

### Supplementary Information

Supplementary Figures.

## Data Availability

Available upon request to the corresponding author.
