# Supplementary material for: Neural mechanism facilitating PM2.5-related cardiac arrhythmias through cardiovascular autonomic and calcium dysregulation in a rat model
Source: Sci Rep. 2023 Sep 25;13:16016. doi: 10.1038/s41598-023-41148-8 (PMC10520066; doi:10.1038/s41598-023-41148-8)
Supplement: Supplementary file 1 — Supplementary Figures. [file 41598_2023_41148_MOESM1_ESM.pdf]

Supplementary materials

Supplemental Figure 1.

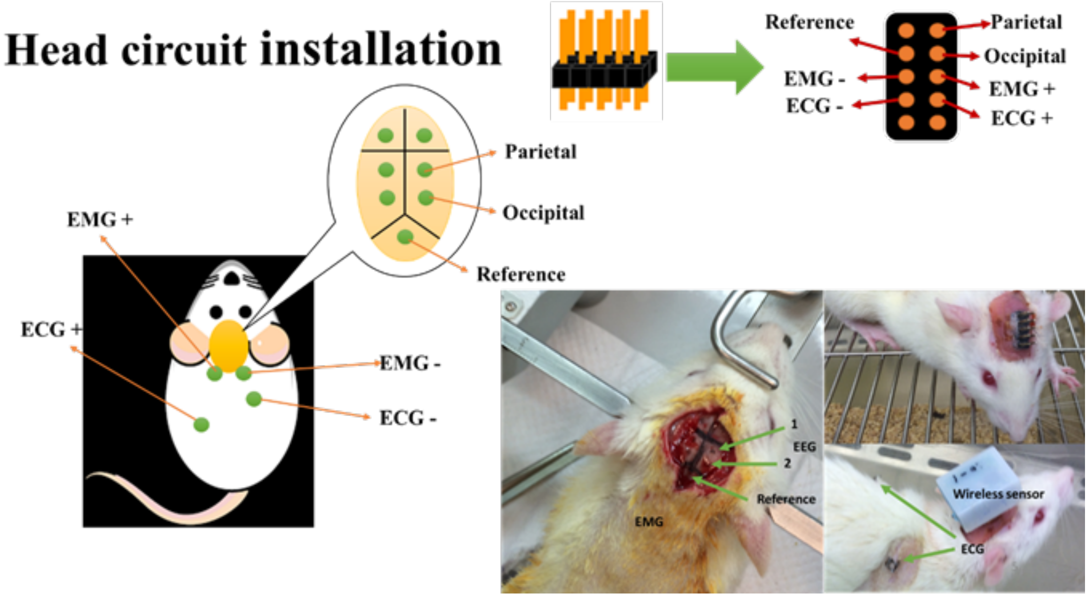

Supplemental Figure 2.

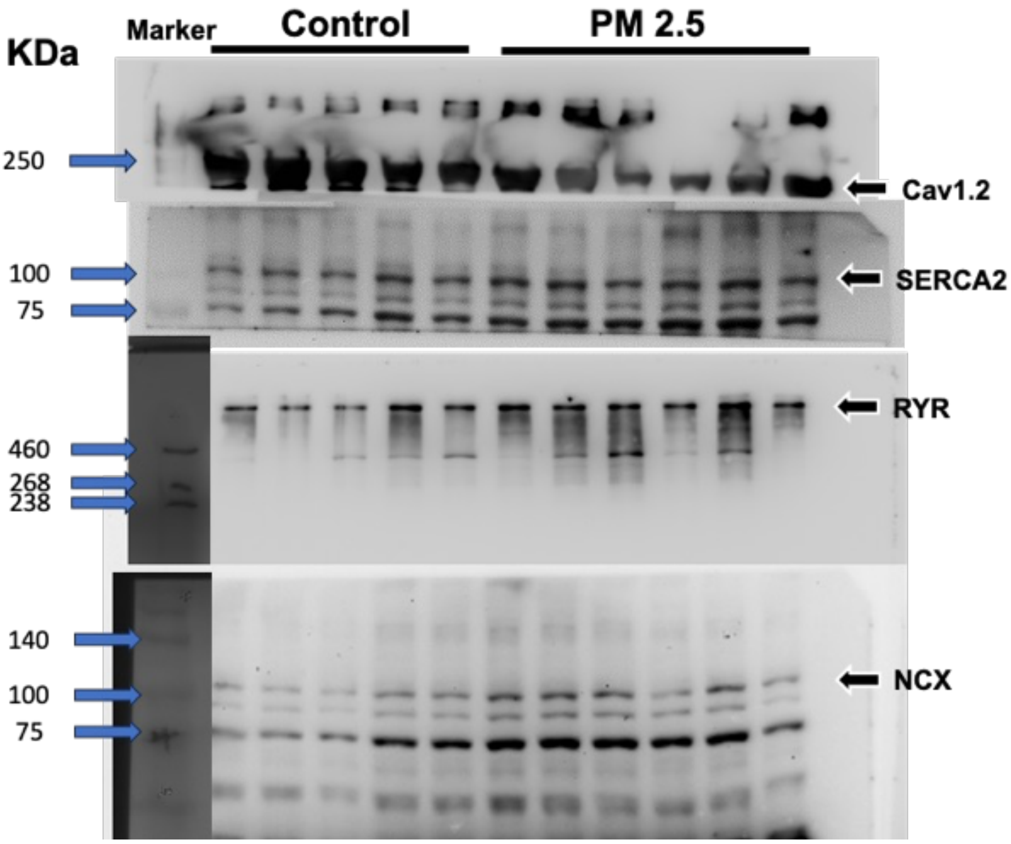

Supplemental Figure 3.

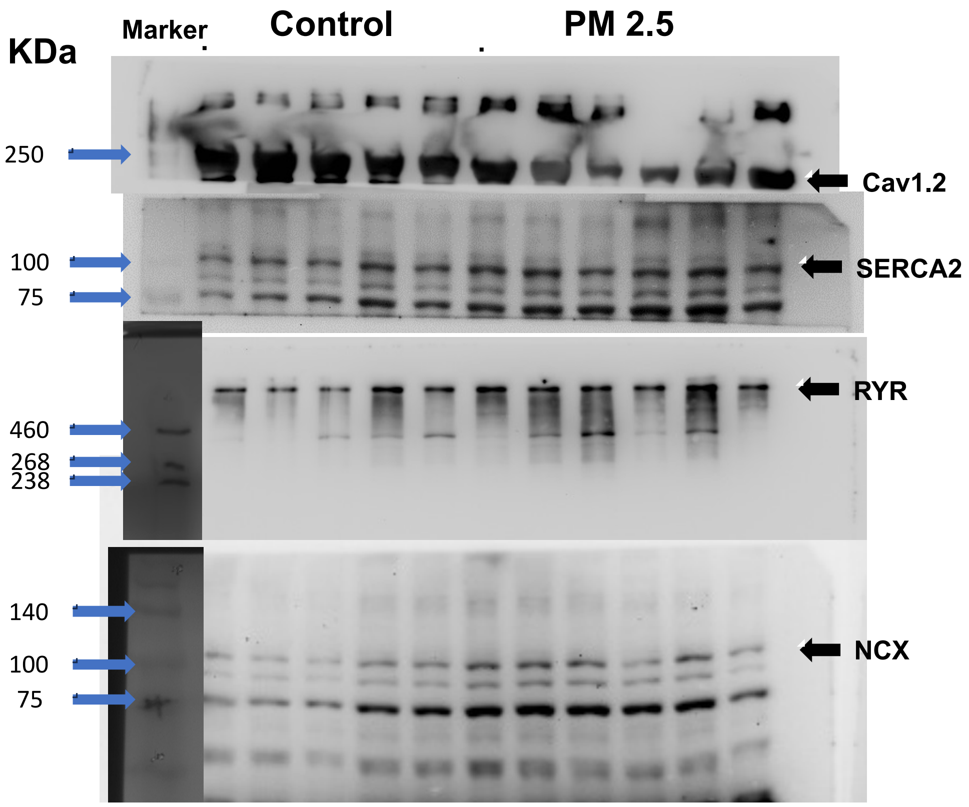

Supplemental Figure 4.

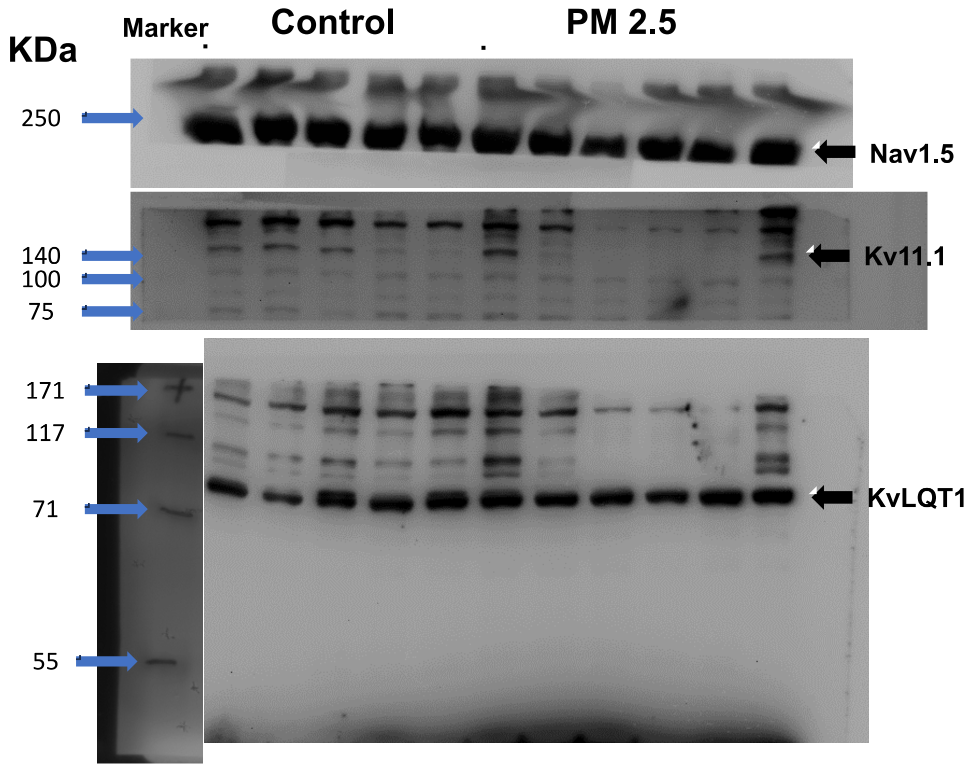

Figure legend:

Supplemental Figure 1. The layout for the installation of EEG head circuit, and body electrode for EMG and ECG.

Supplemental Figure 2. Original Western blot analysis of the ion channel protein expression of the ventricular tissue of the PM2.5 group and the control group. The expression of CaV1.2, SERCA2, RYR, and NCX are shown. KDa= kilodaltons

Supplemental Figure 3. Original Western blot analysis of the ion channel protein expression of the ventricular tissue of the PM2.5 group and the control group. The expression of Nav1.5, Kv11.1, and KvLQT1 are shown.

Supplemental Figure 4. Original Western blot analysis of the ion channel protein expression of the ventricular tissue of the PM2.5 group and the control group. The expression of Kir2.1 is shown.
